# Professionalism among medical residents in a young second-level university in Iran: a cross-sectional study

**DOI:** 10.18502/jmehm.v13i1.2463

**Published:** 2020-02-23

**Authors:** Elaheh Mianehsaz, Seyed Mohammad Reza Tabatabaee, Mohammad Reza Sharif, Hamid Reza Gilasi, Hamid Reza Shojaee Far, Behzad Nejad Tabrizi

**Affiliations:** 1 *Assistant Professor, Department of Physical Medicine and Rehabilitation, Faculty of Medicine, Kashan University of Medical Sciences, Kashan, Iran.*; 2 *Assistant Professor, Department of Emergency Medicine, Faculty of Medicine, Kashan University of Medical Sciences, Kashan, Iran.*; 3 *Professor, Infectious Diseases Research Center, Kashan University of Medical Sciences, Kashan, Iran.*; 4 *Associate Professor, Department of Epidemiology and Biostatistics, Faculty of Health, Kashan University of Medical Sciences, Kashan, Iran.*; 5 *Researcher, Student Research Committee, Kashan University of Medical Sciences, Kashan, Iran.*; 6 *Researcher, Student Research Committee, Kashan University of Medical Sciences, Kashan, Iran.*

**Keywords:** Professionalism, Residency, Surveys, Questionnaires, Iran

## Abstract

Professionalism is a set of behaviors that build trust in physicians’ relationships with patients and the public. The aim of this study was to assess professionalism among residents in Kashan University of Medical Sciences, Kashan, Iran.

This cross-sectional study was conducted on 139 residents recruited through the census method. Data were collected using the American Board of Internal Medicine Professionalism Questionnaire. The first part of the questionnaire was on residents’ personal characteristics, and the second part contained fifteen items in the three domains of professionalism, namely excellence, honor/integrity, and altruism/respect. The mean scores of the questionnaire and its domains were calculated and their relationships with residents’ personal characteristics were evaluated.

The mean scores (± SD) of professionalism and its excellence, honor/integrity, and altruism/respect domains were 4.93 ± 2.4, 5.92 ± 1.85, 4.94 ± 3.39, and 4.35 ± 3.27, respectively (in a range of 0-10). Professionalism had significant relationships only with residents’ specialty and gender.

The level of professionalism in residents was low, which requires the attention of educational authorities. Moreover, the mean score of professionalism among residents in surgical specialties was significantly lower than non-surgical specialties. Various factors can be considered in this regard and it cannot be concluded that the lower score means worse professional behavior.

## Introduction

Medical professionalism is a social contract with the society that builds trust in physicians’ relationships with patients and the public (1). Its essence is the mutual physician-patient trust. The key characteristic of professional physicians is the prioritization of patients’ needs over their own (2, 3). There is no single, universal definition of physician professionalism. Professionalism is commitment to altruism, honesty, confidentiality, excellence, respect for patients' right to autonomy, and having appropriate relationships with them (4).

Medical residents are among the main providers of healthcare services. After graduation, some of them are employed as medical faculty members and become role models for their peers and medical students. It is clear that any problem in physicians’ professional behavior can compromise patients’ health. Several studies showed that medial students and residents who demonstrated limited accountability and professionalism during their university education committed medical errors several times more than their peers after graduation (5 - 7). Therefore, medical residents’ professionalism needs to be continuously monitored and promoted throughout their training program (8). The Accreditation Council for Graduate Medical Education (ACGME) considers professionalism as one of the six core competencies based on which residents are to be regularly evaluated (9).

The importance of the issues of professionalism may be differently ranked and valued by different subspecialties (10, 11). 

There are different instruments for evaluation of professionalism, many of which have not been fully tested for reliability and validity. The most commonly used methods for assessment of professionalism are direct observation, patient assessment, objective structural clinical examination (OSCE), clinical incident report, resident portfolio, professionalism Mini-Evaluation Exercise, videotape analysis and peer assessment (9, 12, 13). In peer assessment, peers with the same professional position who have been in direct unplanned contact with the intended person and have no superiority over him/her are asked to comment on his/her professional practice. This method provides valuable information about professionalism (14, 15). One of the instruments for peer professionalism assessment is the questionnaire developed by the American Board of Internal Medicine (2). This questionnaire was used in previous studies to assess professionalism among physical medicine residents in the United States (16), medical residents in all specialties in two universities in Tehran, Iran (2), and physical medicine and rehabilitation residents in Iran (17). 

All previous studies into medical residents’ professionalism in Iran were conducted in leading universities (2, 18), and hence, there is limited information about medical residents’ professionalism in other universities. The present study aimed to evaluate medical residents’ professionalism in a second-level university, i.e., Kashan University of Medical Sciences, Kashan, Iran. This university was established around thirty years ago and is considered a young university. Most residency programs in this university have a history of less than ten years, and there are no subspecialty fellowship programs at the moment. Professors and senior residents are direct role models for junior students and residents. Therefore, the present study aimed to assess professionalism among residents in this university.

## Methods

This cross-sectional study was conducted between July and September 2017.

Study setting was Kashan University of Medical Sciences, Kashan, Iran, and study population comprised all medical residents in this university, including residents in pediatrics, internal medicine, general surgery, neurology, neurosurgery, gynecology, anesthesia, psychiatry, infectious diseases, pathology, and radiology. All residents in the university were recruited to the study via the census method. They were provided with explanations about the aim of the study and confidentiality of their information, and then, their verbal informed consents were secured. Residents were included if they agreed to participate. 

Data collection tool was the Persian version of the American Board of Internal Medicine (ABIM) Professionalism Questionnaire (2). The reliability and content validity of the questionnaire were confirmed at 0.88 by Aramesh et al. (2). In the present study, the Cronbach's alpha coefficient of the mentioned questionnaire was estimated at 0.878. 

This questionnaire included two main parts. The first part was related to residents’ personal characteristics, namely age, gender, academic year, residency specialty, familiarity with the concept of professionalism, participation in professionalism workshops, and self-study about professionalism. Participants’ specialties were divided into the main two categories of non-surgical specialties (consisting of general surgery, neurosurgery, gynecology, and anesthesia) and surgical specialties (consisting of pediatrics, internal medicine, neurology, psychiatry, infectious diseases, pathology, and radiology). The second part of the questionnaire contained fifteen items on professional behaviors in three domains, namely excellence (four items), honor/integrity (four items), and altruism/respect (seven items). The items of the excellence domain (items 1 - 4) assessed residents’ beliefs about the availability of good role models in the areas of professional conduct, patient education, and student training. The honor/integrity domain (items 5 - 8) dealt with residents’ belief in their peers’ honesty and avoidance of nonprofessional practice. For instance, items in this domain were related to the possibility of lying to patients and asking junior residents to extract data from patients’ medical records. The altruism/respect domain (items 9 - 15) had items on residents’ respect for patients, peers, and hospital regulations, avoidance of wasting hospital resources, and consideration of patients’ convenience in performing diagnostic and medical procedures. Items were scored from 0 (i.e., “Lowest level of professionalism”) to 10 (i.e., “Highest level of professionalism”). Thus, the possible total score of the questionnaire was 0 - 150, and the possible total scores of its three domains were 0 - 40, 0 - 40, and 0 - 70, respectively. In order to generate a uniform 0 - 10 scoring scale for all domains, the total score of each domain was divided by the number of its items. 

After obtaining the legal and ethical approvals from Kashan University of Medical Sciences, Kashan, Iran, (code: IR.KAUMS.REC.1394.146), two research assistants (medical interns who were well aware of the study aims) provided the study questionnaires to participants and asked them to complete them at their convenience. Some participants completed and returned their questionnaires on the same day, while others returned their questionnaires days afterwards. Participants were reminded to complete the questionnaires through follow-up personal contact or telephone calls.

***Data Analysis***

Study data were analyzed using the SPSS program (v. 21.0). The independent-sample t-test was conducted to compare professionalism scores based on participants’ characteristics, and it had two levels. Similarly, the one-way analysis of variance or the Kruskal-Wallis test was performed for characteristics with three or more levels. The level of significance was set at less than 0.05.

## Results

Among all 181 residents in the study setting, four refused to participate and seventeen were in other universities in Iran as guest students. Thus, 160 residents participated in the study. Despite frequent follow-up contacts, twelve residents did not return their questionnaires. Moreover, nine questionnaires were partially filled out. Thus, 139 questionnaires were entered in the final data analysis (a response rate of 86.87%). Around 73% of the participants were familiar with the concept of professionalism, 11.5% had attended professional ethics or professionalism workshops or courses, and 24% had had self-study in this area. 

Participants’ mean score of professionalism was 73.93 ± 36.01 (in the possible range of 0 - 150). The total mean scores of professionalism and its excellence, honor/integrity, and altruism/respect domains on the 0 - 10 scale were 4.93 ± 2.4, 5.92 ± 1.85, 4.94 ± 3.39, and 4.35 ± 3.27, respectively.

The mean score of professionalism was significantly greater among residents in non-surgical specialties than in surgical specialties (5.64 ± 2.40 vs. 3.54 ± 1.62; *P* < 0.001). Moreover, female participants’ mean score of professionalism was significantly greater than their male counterparts (*P* = 0.006). However, there was no significant relationship between the mean score of professionalism and the other personal characteristics of participants (*P* > 0.05; Table 1).

**Table 1:** The mean scores of professionalisms and its domains based on participants’ personal characteristics

Professionalism Characteristics		N (%)	Total score	P-value	Excellence	P value	Honor/ Integrity	P value	Altruism / Respect	P value
Gender	Male	67 (47.97)	4.35±2.16	0.006	5.73±1.92	0.24	4.01±3.24	0.22	3.57±2.90	0.001*
	Female	72 (52.03)	5.47±2.50		6.10±1.77		5.79±3.32		4.91±3.49	
Marital status	Single	43 (31.08)	4.94±2.30	0.94	5.58±1.95	0.23	5.34±3.09	0.33	4.35±3.26	0.95*
	Married	96 (68.92)	4.89±2.44		6.06±1.80		4.73±3.51		4.32±3.28	
Specialty	Non-surgical	91 (65.54)	5.64±2.43	<0.0001	5.93±1.97	0.96	5.81±3.48	>0.0001	5.37±3.26	0.0001*
	Surgical	48 (34.46)	3.54±1.62		5.91±1.60		3.22±2.46		2.37±2.19	
Academic year	First	46 (33.11)	4.91±2.62	0.42	5.51±1.84	0.13	4.85±3.34	0.28	4.60±3.4	0.27†
	Second	33 (23.65)	5.48±2.40		5.83±1.83		5.86±3.38		5.05±3.32	
	Third	28 (20.27)	4.66±2.27		6.59±1.79		4.53±3.26		3.62±3.25	
	Fourth	32 (22.97)	4.59±2.17		5.92±1.85		4.39±3.55		3.91±2.96	
Familiarity with professionalism	Yes	101 (72.97)	4.79±2.40	0.24	6.01±1.86	0.29	4.75±3.44	0.28	4.1±3.28	0.14*
	No	38 (27.03)	5.31±2.38		5.67±1.83		5.45±3.25		5.03±3.16	
Participation in workshops	Yes	16 (11.49)	4.75±2.23	0.77	5.77±2.07	0.67	4.83±3.55	0.58	4.32±3.08	0.97#
	No	123 (88.51)	4.95±2.43		5.94±1.83		4.99±3.38		4.36±3.29	
Self-study on professionalism	Yes	33 (23.65)	4.69±2.57	0.53	5.12±2.23	0.01	4.66±3.56	0.60	4.45±3.42	0.85*
	No	106 (76.35)	5±2.36		6.17±1.66		5.02±3.35		4.32±3.24	
Number of learning hours per week	50–60	47 (33.78)	4.87±2.51	0.38	5.86±1.79	0.16	4.91±3.45	0.79	4.28±3.33	0.68^
	61–70	33 (24.32)	4.73±2.14		5.30±2.23		4.49±3.17		3.76±3.08	
	71–80	25 (17.57)	5.29±2.61		6.52±1.94		5.20±3.64		4.65±3.63	
	81–90	9 (6.76)	6.15±2.05		6.82±1.45		6.22±2.96		5.73±2.87	
	> 90	25 (17.57)	4.89±2.45		5.99±1.19		4.70±3.60		4.38±3.31	

* *: The results of the independent-sample t-test;*

† *: The results of the one-way analysis of variance;*

^ *: The results of the Kruskal-Wallis test;*

# *: The results of the Mann-Whitney U test*

## Discussion

This study evaluated professionalism among medical residents in Kashan University of Medical Sciences, Kashan, Iran. The total mean score of professionalism was 73.93 ± 36.01 on the 0 - 150 scale, and 4.93 ± 2.4 on the 0 - 10 scale. A study on residents in Tehran and Shahid Beheshti Universities of Medical Sciences, Tehran, Iran, reported a professionalism mean score of 6.12 ± 0.37, indicating moderate professionalism (2). All these findings may demonstrate the ineffectiveness of the interventions that have been implemented for promotion of professionalism in recent years. 

Other studies on physical medicine and rehabilitation residents throughout the United States and Iran found that their mean scores of professionalism were 7.7 and 7.67, respectively (16, 17). The significantly higher total professionalism scores in those studies compared to the present study may be due to the fact that they were conducted on physical medicine and rehabilitation residents who did not do night shifts and did not attend the operating room, while samples of the present study consisted of residents from different specialties and different work shifts. 

Our findings also showed a significant relationship between the type of specialty and the total mean score of professionalism, so that residents in non-surgical specialties obtained significantly greater scores than their counterparts in surgical specialties. High occupational stress, emergency situations, high frequency of night shifts, lack of sleep, lack of adequate time for self-study, and role multiplicity might have contributed to the lower professionalism mean score among residents in surgical specialties. Further studies are needed to determine the factors affecting professionalism. 

The mean scores of the excellence, honor/integrity, and altruism/respect domains of professionalism in the present study were 5.92 ± 1.85, 4.94 ± 3.39, and 4.35 ± 3.27, respectively. Thus, the highest and the lowest scores were related to the excellence and the altruism/respect domains, respectively. These findings indicate that participants’ practice was better in terms of prioritizing patients’ needs over their own needs, attempting to broaden their knowledge, and training junior residents compared to their practice in terms of respecting hospital regulations, saving resources, and ensuring patients’ convenience. Further studies are needed to provide reasons for such findings. 

Our findings also showed that residents in non-surgical specialties obtained significantly greater professional scores than those in surgical specialties (5.64 ± 2.40 vs. 3.54 ± 1.62; *P* < 0.001). However, we could not find any study in this area for the purpose of comparison. This finding may be due to the fact that residents in surgical specialties worked in the stressful environment of operating rooms and needed to make prompt clinical decisions in emergency conditions. 

Another finding of the present study was the significantly greater professionalism mean score among female participants compared to their male counterparts. However, there was no significant relationship between professional mean score and the other personal characteristics of participants. Similarly, a former study indicated no significant relationship between residents’ professionalism and their personal characteristics (17).

Findings also indicated that around 73% of the participants were familiar with the concept of professionalism, and 24% had had self-study about it. A study on medical students in Shiraz, Iran, showed that only 40% of them were familiar with the concept (18), and another study reported that only 10% of the residents had had self-study in this area (17). Personal attitudes towards professionalism, self-study on the subject, and participation in conferences and workshops can affect residents’ perceptions of their peers’ professionalism. In other words, residents with greater knowledge about professionalism more seriously expect their peers to adhere to the principles of professional practice and may therefore score their peers’ professionalism more strictly and cautiously. Similarly, our findings showed that residents who were familiar with the subject of professionalism, had had self-study in this area, or had participated in relevant workshops evaluated their peers’ professionalism more poorly. Of course, the difference was not statistically significant.

## Conclusion

Based on the results, the medical residents in this study had a low level of professionalism. The high score of the excellence domain and the low score of the honor/integrity and altruism/respect domain denote that the residents had a better condition in prioritization of the needs of patients over their own interests, trying to be scientifically updated, and improving education quality compared to respecting hospital laws, avoiding resource loss, and paying attention to patients’ comfort in the treatment procedures.

The findings showed that residents in non-surgical specialties obtained significantly greater professional scores than those in surgical specialties. This may be due to the fact that residents in surgical specialties worked in the stressful environment of operating rooms and needed to make prompt clinical decisions in emergency conditions.

## Recommendations

We evaluated the relationship between professionalism and some personal characteristics such as the type of specialty, gender, and marital status. It is recommended that future studies evaluate the relationship between professionalism and other factors such as patients’ conditions, faculty-resident ratio, patient-resident ratio, and workload. Moreover, we found that despite having good role models, residents believed that their peers had poor professional conduct. Thus, studies are needed to evaluate the reasons behind the insignificant effects of role models on residents’ professional conduct. Moreover, only 11.5% of the participants had participated in professionalism and professional ethics workshops and courses; thus, studies are needed to evaluate the effects of such workshops on medical residents’ professionalism. 

## Citation to this article:

Mianehsaz E, Tabatabaee SMR, Sharif MR, Gilasi HR, Shojaee Far HR, Nejad Tabrizi B. Professionalism among medical residents in a young second-level university in Iran: a cross-sectional study. J Med Ethics Hist Med. 2020; 13: 1.

## References

[1] Büke AS, Öztürkçü OSK, Yılmaz Y, Sayek I (2018). Core professionalism education in surgery: a systematic review. Balkan Med J.

[2] Aramesh K, Mohebbi M, Jessri M, Sanagou M (2009). Measuring professionalism in residency training programs in Iran. Med Teach.

[3] Lynch DC, Surdyk PM, Eiser AR (2004). Assessing professionalism: a review of the Literature. Medical Teacher.

[4] Asghari F, Nikavaran Fard N, Atabaki A (2011). Are we proper role models for students? Interns' perception of faculty and residents' professional behavior. Postgraduate Medical Journal.

[5] Papadakis MA, Teherani A, Banch M (2005). Disciplinary action by medical boards and prior behaviour in medical school. N Engl J Med..

[6] Papadakis MA, Hodgson CS, Teherani A, Kohatsu ND (2004). Unprofessional behavior in medical school is associated with subsequent disciplinary action by a state medical board. Acad Med.

[7] Papadakis MA, Arnold GK, Blank LL, Holmboe ES, Lipner RS (2008). Performance during internal medicine residency training and subsequent disciplinary action by state licensing boards. Ann Intern Med.

[8] Conran RM, Powell SZ, Domen RE (2018). Development of professionalism in graduate medical education: a case-based educational approach from the college of American pathologists' graduate medical education committee. Acad Pathol.

[9] Lee FY, Yang YY, Hsu HC (2011). Clinical instructors' perception of a faculty development programme promoting postgraduate year-1 (PGY1) residents' ACGME six core competencies: a 2-year study. BMJ Open.

[10] Domen RE, Talbert ML, Johnson K (2015). Assessment and management of professionalism issues in pathology residency training: results from surveys and a workshop by the graduate medical education committee of the college of American pathologists. Acad Pathol.

[11] Hoonpongsimanont W, Sahota PK, Chen Y (2018). Physician professionalism: definition from a generation perspective. International Journal of Medical Education..

[12] Malakoff GL, Payne CL, Staton LJ, Kolade VO, Panda M (2014). Accounting for professionalism: an innovative point system to assess resident professionalism. J Community Hosp Intern Med Perspect.

[13] Goldie J (2013). Assessment of professionalism: a consolidation of current thinking. Med Teach.

[14] Bryan RE, Krych AJ, Carmichael SW, Viggiano TR, Pawlina W (2005). Assessing professionalism in early medical education: experience with peer evaluation and self-evaluation in the gross anatomy course. Ann Acad Med Singapore.

[15] Arnold L, Shue CK, Kalishman S (2007). Can there be a single system for peer assessment of professionalism among medical students? A multi-institutional study. Acad Med.

[16] DeLisa JA, Foye PM, Jain SS, Kirshblum S, Christodoulou C (2001). Measuring professionalism in a physiatry residency training program. American Journal of Physical Medicine and Rehabilitation.

[17] Ahadi T, Mianehsaz E, Raissi G, Moraveji SA, Sharifi V (2015). Professionalism in residents of physical medicine and rehabilitation in Iran. J Med Ethics Hist Med..

[18] Askarian M, Ebrahimi Nia MJ, Sadeghipur F, Danaei M, Momeni M (2015). Shiraz medical students' perceptions of their colleagues' professional behavior. J Adv Med Educ Prof.

